# The β_2_-Subunit of Voltage-Gated Calcium Channels Regulates Cardiomyocyte Hypertrophy

**DOI:** 10.3389/fcvm.2021.704657

**Published:** 2021-07-07

**Authors:** Simone Pickel, Yiliam Cruz-Garcia, Sandra Bandleon, Katalin Barkovits, Cornelia Heindl, Katharina Völker, Marco Abeßer, Kathy Pfeiffer, Alice Schaaf, Katrin Marcus, Petra Eder-Negrin, Michaela Kuhn, Erick Miranda-Laferte

**Affiliations:** ^1^Institute of Physiology, University of Würzburg, Würzburg, Germany; ^2^Comprehensive Heart Failure Center, University Hospital Würzburg, Würzburg, Germany; ^3^Medizinisches Proteom-Center, Medical Faculty, Ruhr-University Bochum, Bochum, Germany; ^4^Medical Proteome Analysis, Center for Proteindiagnostics (PRODI), Ruhr-University Bochum, Bochum, Germany; ^5^Institut für Biologische Informationsprozesse, Molekular- und Zellphysiologie (IBI-1), Forschungszentrum Jülich, Jülich, Germany

**Keywords:** L-type voltage-gated calcium channels, cardiac hypertrophy, calpain, cardiomyocytes, calpastatin, Ca_v_β_2_-subunit

## Abstract

L-type voltage-gated calcium channels (LTCCs) regulate crucial physiological processes in the heart. They are composed of the Ca_v_α_1_ pore-forming subunit and the accessory subunits Ca_v_β, Ca_v_α_2_δ, and Ca_v_γ. Ca_v_β is a cytosolic protein that regulates channel trafficking and activity, but it also exerts other LTCC-independent functions. Cardiac hypertrophy, a relevant risk factor for the development of congestive heart failure, depends on the activation of calcium-dependent pro-hypertrophic signaling cascades. Here, by using shRNA-mediated Ca_v_β silencing, we demonstrate that Ca_v_β_2_ downregulation enhances α1-adrenergic receptor agonist-induced cardiomyocyte hypertrophy. We report that a pool of Ca_v_β_2_ is targeted to the nucleus in cardiomyocytes and that the expression of this nuclear fraction decreases during *in vitro* and *in vivo* induction of cardiac hypertrophy. Moreover, the overexpression of nucleus-targeted Ca_v_β_2_ in cardiomyocytes inhibits *in vitro*-induced hypertrophy. Quantitative proteomic analyses showed that Ca_v_β_2_ knockdown leads to changes in the expression of diverse myocyte proteins, including reduction of calpastatin, an endogenous inhibitor of the calcium-dependent protease calpain. Accordingly, Ca_v_β_2_-downregulated cardiomyocytes had a 2-fold increase in calpain activity as compared to control cells. Furthermore, inhibition of calpain activity in Ca_v_β_2_-downregulated cells abolished the enhanced α1-adrenergic receptor agonist-induced hypertrophy observed in these cells. Our findings indicate that in cardiomyocytes, a nuclear pool of Ca_v_β_2_ participates in cellular functions that are independent of LTCC activity. They also indicate that a downregulation of nuclear Ca_v_β_2_ during cardiomyocyte hypertrophy promotes the activation of calpain-dependent hypertrophic pathways.

## Introduction

L-type voltage-gated calcium channels (LTCCs) are heteromultimeric proteins composed of the Ca_v_α_1_ pore-forming subunit and the accessory subunits Ca_v_β, Ca_v_α_2_δ, and Ca_v_γ. LTCCs are responsible for Ca^2+^ influx into cardiomyocytes following plasma membrane depolarization. Ca^2+^ entry through these channels regulates crucial processes, including duration, and amplitude of the action potentials, excitation-contraction coupling, and gene expression (1).

The Ca_v_β subunit, a member of the MAGUK-scaffolding protein family, is a cytosolic soluble protein that binds to the channel with high affinity. Of the four Ca_v_β isoforms (Ca_v_β_1_−β_4_), Ca_v_β_2_ is the predominant one in murine and human cardiomyocytes (2, 3) and mutations in the Ca_v_β_2_ gene have been associated with cardiac arrhythmias and sudden death (4). There are five splice variants of Ca_v_β_2_ (Ca_v_β_2a_-Ca_v_β_2e_), which differ only in the N-terminus (3, 5).

The physiological role of Ca_v_β has been assessed by several groups in different experimental models. In heterologous expression systems, Ca_v_β regulates LTCC membrane trafficking and Ca^2+^ currents (6, 7). In adult ventricular cardiomyocytes, a disruption of the Ca_v_1.2-Ca_v_β association affects the inactivation rate of LTCC (8). In mice, global knockout of Ca_v_β_2_ produces a lethal phenotype at early embryonic stages due to impaired cardiac development and contractile dysfunction (9). However, conditional cardiomyocyte-specific downregulation of the Ca_v_β_2_ gene in adult mice only caused a small reduction in Ca^2+^ currents without altering cardiac mechanical functions, at least under resting, physiological conditions (2). Additionally, murine cardiomyocytes overexpressing dihydropyridine (DHP)-resistant recombinant Ca_v_1.2 channels lacking key amino acids necessary for Ca_v_β binding, displayed normal Ca^2+^ currents in the presence of DHP (10). Due to these contradictory results, the regulatory role of Ca_v_β_2_ in LTCC activity in cardiomyocytes remains controversial. In recent years, our own and other published studies have demonstrated that in neurons, skeletal muscle cells and heterologous expression systems, Ca_v_β instead participates in and controls other cellular processes such as endocytosis and gene expression, without directly interacting with LTCCs (11–13). These results suggested that Ca_v_β could regulate cardiomyocyte functions independently of LTCCs activity.

Cardiac hypertrophy is a relevant risk factor for the development of congestive heart failure and it is usually driven by calcium-dependent pro-hypertrophic signaling cascades. However, the contribution Ca_v_β functions on cardiac hypertrophy has not been fully addressed. Here, we have dissected the role of Ca_v_β_2_ in cardiomyocyte hypertrophy by using shRNA-mediated Ca_v_β_2_ knockdown in neonatal rat cardiomyocytes (NRCs). We provide evidence that Ca_v_β_2_ controls the expression of calpastatin and thereby the activity of calpain, a pro-hypertrophic Ca^2+^-dependent protease (14, 15). Moreover, we reveal that a fraction of Ca_v_β_2_ is targeted to the nucleus of cardiomyocytes and that this pool decreases during cardiac hypertrophy. Finally, we also demonstrate that nucleus-targeted Ca_v_β_2_ can attenuate cardiomyocyte hypertrophy.

## Materials and Methods

### Isolation and Culture of Neonatal Rat Cardiomyocytes

Neonatal rat cardiomyocytes were isolated as previously described by Kirschmer et al. (16). Briefly, for each replicate of an experiment, 10–15 pups (1–3 day old) from 2 to 3 Wistar rats (Charles River Laboratory) were euthanized by decapitation and the hearts were cut into small pieces in a Petri dish containing calcium- and bicarbonate-free Hank's balanced salt solution with Hepes (CBFHBSS) (137 mM NaCl, 5.36 mM KCl, 0.81 mM MgSO_4_, 5.55 mM D-Glucose, 0.44 mM KH_2_PO_4_, 0.34 mM Na_2_HPO_4_, 20 mM Hepes, pH 7.4) supplemented with penicillin-streptomycin. The heart pieces were incubated for 15 min at 37°C with 20 ml of CBFHBSS supplemented with 15 μg/ml of DNAase (Sigma Aldrich) and 0.1 mg/ml/heart of Trypsin (Sigma Aldrich). After incubation, fresh enzyme solution preheated to 37°C was added, the samples were reincubated for 10 min at 37°C, and the heart pieces were allowed to settle for 3 min. The supernatant was then transferred to a new tube containing fetal calf serum (FCS). The last two-steps were repeated until the heart pieces were completely digested. After the digestion, the cells were centrifuged at 500 × g for 5 min. The pellet was then re-suspended in minimal essential medium (MEM) with 5% FCS. The homogenate was filtered through a 0.22 μm sterile filter, plated in 100-mm dishes and incubated for 45–60 min at 37°C. During this period, the fibroblasts settled down and fixed to the plate, whereas the cardiomyocytes remained in the supernatant. After the incubation time, the supernatant was collected and cardiomyocytes were counted using an automated cell counter. For biochemical studies, 2 × 10^6^ cells per well were seeded into 6-well plates. For fluorescence microscopy and the measurement of Ca^2+^ transients, 2 × 10^5^ cells were plated on laminin-coated slides. The cardiomyocytes were kept in culture for 6 days. Initially, the cells were incubated with MEM supplemented with 5% FCS during 2 days. On day 3, the cells were transduced with the corresponding adenovirus at the indicated multiplicity of infection (MOI) and kept on serum-free MEM for 4 h. Cells were then washed with phosphate-buffered solution (PBS; 137 mM NaCl, 2.7 mM KCl, 2 mM KH_2_PO_4_, 8 mM Na_2_HPO_4_, pH 7.4) and fresh 1% FCS-MEM medium was added, and the plates were incubated for 24 h. For the evaluation of agonist-induced hypertrophy, cells were serum starved on day 4 and stimulated for 24 h on day 5 with phenylephrine (PE) (50 μM), or PE plus calpeptin (25 μM). Cells treated with vehicle were used as negative control.

### Calcium Measurements

The measurement of the fluorometric Ca^2+^ transients in NRCs was performed as described by Kirschmer et al. (16). Briefly, cells were cultured on cover slides coated with laminin (Roche) and loaded for 20 min at room temperature with Fura-2 (2 mM) in a Ca^2+^-free normal Tyrode solution (140 mM NaCl, 4 mM KCl, 1 mM MgCl_2_, 5 mM Hepes, 10 mM glucose, pH 7.4). Ca^2+^ transients were measured in normal Tyrode solution supplemented with 1 mM CaCl_2_ at a pacing frequency of 1 Hz using the “Myocyte and Contractility System” from Ionoptix. Data were corrected for background fluorescence 340/380 and analyzed using the IonWizard 6.3 software (Ionoptix).

### Calpain Activity Assay

Calpain activity was evaluated using the Calpain-Glo^TM^ protease assay (Promega). NRCs were cultured in 6-well plates. After adenoviral transduction and agonist-induced cardiac hypertrophy, cultures were incubated with 40 μM Suc-LLVY-Glo^TM^ substrate for 1 h at 37°C. Cells were washed with PBS and lysed with Glo Lysis buffer (Promega) containing 1 × halt protease inhibitor cocktail (Thermo Fisher Scientific) and 25 μM calpeptin. Three replicas of each cell lysate were diluted 1:1 with the calpain-Glo^TM^ buffer containing the luciferin detection reagent. Following 5 min of incubation at room temperature, the luciferase activity was measured in a 96-well plate using a Victor2 microplate multilabel reader (Perkin Elmer).

### Molecular Biology

The expression of the different Ca_v_β isoforms and Ca_v_β_2_ splice variants in NRCs was determined by RT-PCR. After isolation of total RNA from NRC using a TRIzol™-based method, cDNA were synthetized following the instructions of the QuantiTect® Reverse Transcription Kit (Qiagen). RT-PCR were performed using the following primers: Ca_v_β_1_ forward primer 5′- TACACGAGCCGGCCGTCAGACTCCG−3′, Ca_v_β_1_ reverse primer 5′-GCAGGCGAAGGCTGTCCAGTTTGAC-3′, Ca_v_β_2_ forward primer 5′- GAGTCACTGCTGACATCTCCCTGGC-3′, Ca_v_β_2_ reverse primer 5′- TCCAGATAGTCGGCGAGATGCTCAC-3′, Ca_v_β_3_ forward primer 5′- CCAAGCGCTCTGTGCTCAACAATC-3′, Ca_v_β_3_ reverse primer 5′- GGGACTTCCCCCTGGAGCGGATC-3′, Ca_v_β_4_ forward primer 5′- AATGTGAGCTACTGTGGTGCCCTG-3′, Ca_v_β_4_ reverse primer 5′- CTCCCAGACTGGAGGAAGAGTTTCC-3′, Ca_v_β_2a_ forward primer 5′- ATGCAGTGCTGCGGGCTGG-3′, Ca_v_β_2b_ forward primer 5′- ATGCTTGACAGGCAGTTGGTGTCTTC-3, Ca_v_β_2c_ forward primer 5′- ATGGACCAGGCGAGTGGACTGG-3, Ca_v_β_2d_ forward primer 5′- TGATGACATCTGTATCTGGCAAACCAG-3, Ca_v_β_2e_ forward primer 5′- ATGAAGGCCACCTGGATCAGGC−3 and the common reverse primer for all the Ca_v_β_2_ splice variants 5′- TCTTTAACCAGCCGTCCTATCCACC−3′. cDNA from adult brain tissue was used as control for the RT-PCR reactions.

For Ca_v_β_2b_ (Accession Number XM_006254303) short hairpin RNA (shRNA)-mediated knockdown in NRCs, appropriate complementary single-stranded DNA oligonucleotides were designed using the Block-iT^TM^ RNAi designer platform (Thermo Fisher Scientific). Complementary oligonucleotides for each construct were annealed at 55°C and cloned, according to the manufacturer's instructions, into the pENTR^TM^/U6 entry vector using the Block-iT^TM^ U6 RNAi entry vector kit (Thermo Fisher Scientific). The sequences of the complementary single-stranded DNA primers used were the following: shRNA18 forward primer CACCGGTGTCTTCTCAGACTCAATCCGAAGATTGAGTCTGAGAAGACACC, shRNA18 reverse primer AAAAGGTGTCTTCTCAGACTCAATCTTCGGATTGAGTCTGAGAAGACACC, shRNA338 forward primer CACCGCTGTGAAATCGGATTTATTCCGAAGAATAAATCCGATTTCACAGC, shRNA338 reverse primer AAAAGCTGTGAAATCGGATTTATTCTTCGGAATAAATCCGATTTCACAGC, shRNA508 forward primer CACCGCTATAGACATAGATGCTACTCGAAAGTAGCATCTATGTCTATAGC, shRNA508 reverse primer AAAAGCTATAGACATAGATGCTACTTTCGAGTAGCATCTATGTCTATAGC, shRNA892 forward primer CACCGCGGAAGTTCAGAGTGAAATTCGAAAATTTCACTCTGAACTTCCGC, shRNA892 reverse primer AAAAGCGGAAGTTCAGAGTGAAATTTTCGAATTTCACTCTGAACTTCCGC, scrambled shRNA forward primer CACCGGTCTTCGACTTCATGCAATCCGAAGATTGAGTCTGAGAAGACACC, scrambled shRNA reverse primer AAAAGGTGTCTTCTCAGACTCAATCTTCGGATTGCATGAAGTCGAAGACC. Using that strategy, the following plasmids were obtained: pENTR/U6-shRNA18, pENTR/U6-shRNA338, pENTR/U6-shRNA508, pENTR/U6-shRNA892 and pENTR/U6-scrambled shRNA.

For protein overexpression, the YFP sequence of the vector pEYFP-C1 (Clontech) was amplified by PCR and cloned into the pENTR3C vector (Thermo Fisher Scientific) to produce the pENTR3C-YFP plasmid. To generate the plasmid pENTR3C-Ca_v_β_2b_-YFP, the rat Ca_v_β_2b_ gene (Accession Number XM_006254303) from the pcDNA3.1(-) Ca_v_β_2b_-CFP vector (17) was amplified by PCR and this fragment was inserted in-frame upstream of the YFP sequence into the pENTR3C-YFP plasmid using standard overlapping PCR methods. The pENTR3C-NLS- Ca_v_β_2b_-YFP plasmid was produced by inserting the nuclear localization signal (NLS) of the SV40 large T antigen (PPKKKRKV) at the N-terminus of Ca_v_β_2b_ in the pENTR3C-Ca_v_β_2b_-YFP vector using standard overlapping PCR methods. To generate the pENTR3C-NLS^K4T^-Ca_v_β_2b_-YFP plasmid, the point mutation (Lys4Thr) was introduced into the NLS sequence of the pENTR3C-NLS-Ca_v_β_2b_-YFP vector using standard overlapping PCR methods.

For qRT-PCR analyses of calpastatin expression, cDNA from control and Ca_v_β_2_-downregulated NRCs were prepared as previously described. The qRT-PCR were performed on a LightCycler® 96 (Roche) using the forward primer 5′- GAGAAAACAAAGGATTCCTCCA-3′, the, reverse primer 5′- CTTCATCCACCTTTGGCTTG-3′ and the probe 6 of the Universal ProbeLibrary (Roche). For each run, a standard curve in a range of 10–10^−6^ pg of cDNA was performed to evaluate the efficiency of the qRT-PCR. Efficiencies of the PCR ranging between 80 and 100% were considered as good. A negative control with water instead of cDNA was also included. The expression of the reference gene Rpl 13 was measured with the same cDNA as calpastatin to normalize the data.

For analyses of the expression of the Ca_v_β_2_ splice variants by qRT-PCR, cDNA were prepared as previously described, but using as template RNA extracted from the hearts of sham- and TAC-operated mice. The qRT-PCR were performed as described for calpastatin, but using the probe 16 of the Universal ProbeLibrary (Roche) and the following primers: Ca_v_β_2a_ forward primer 5′- GCGAGTACGGGTGTCCTATGGTTC-3′, Ca_v_β_2b_ forward primer 5′- CTCAATCCAGTATTCCTGGGGGTTC-3, Ca_v_β_2c−d_ forward primer 5′- AACAGTTTTGTCCGCCAGGGTTC-3, Ca_v_β_2e_ forward primer 5′- CTGAAGAGTTCGGACATCTGTGGTTC-3 and the reverse primer 5′- CTGCCGCTCAGCTTCTCTAC-3′. A standard curve in a range of 10–10^−6^ pg of cDNA was performed to evaluate the efficiency of the qRT-PCR. All the qRT-PCR performed to detect the expression of the Ca_v_β_2_ splice variants had an efficiency ranging between 90 and 95%. This allows us to perform an absolute quantification of the expression of each Ca_v_β_2_ splice variant. To normalize the data, the expression of the reference gene GAPDH was measured with the same cDNA as the Ca_v_β_2_ splice variants.

### Preparation of Adenovirus

For Ca_v_β_2b_ knockdown in NRCs, recombinant adenoviral vectors were produced by homologous DNA recombination between each shRNA-containing pENTR/U6 plasmid and the pAD/Block-iT^TM^-Dest vector. The BLOCK-iT™ Adenoviral RNAi Expression System (Thermo Fisher Scientific) was used and the manufacturer's instructions were followed. Adenoviral vectors for the overexpression of proteins under the control of the CMV promoter were generated by homologous DNA recombination between each pENTR3C plasmid and the pAD/CMV/V5-Dest vector, using the pAd/CMV/V5-DEST™ Gateway® Vectors (Thermo Fisher Scientific) according to the manufacturer's instructions. Adenoviral vectors obtained by DNA recombination were linearized with the PacI restriction enzyme. HEK293A cells plated in 60-mm dishes at 70–80% confluence were transfected with 5 μg of each vector using the X-tremeGENE HP DNA Transfection Reagent (Roche). After 10–15 days, when ~80–90% of the cells were lysed, the adenovirus-containing cells were harvested from the culture supernatant. To release the viral particles, harvested cells were lysed by three freeze-thaw cycles of 30 min at −80°C followed by 15 min at 37°C. The primary adenoviral stocks were centrifuged at 3,000 rpm for 15 min at room temperature and the supernatants containing the adenovirus were stored at −80°C. For amplification of the adenoviral stocks, HEK293A cells were seeded into 150-mm dishes at 80–90% confluence and infected with the primary adenoviral stocks. After 2–5 days, when 80–90% of the cells were lysed, the same procedure used to obtain the primary adenoviral stocks was followed. Final viral stocks were concentrated using Amicon®Ultra centrifugal filter units with a 100 000 molecular weight cut-off (Sigma Aldrich) and titrated as described by Baer and Kehn-Hall (18).

### Transverse Aortic Constriction

Left ventricular hypertrophy was induced by transverse aortic constriction (TAC) in 6-week-old male C57Bl6 mice (*N* = 3) as described by Klaiber et al. (19). The corresponding sham-operated animals (*N* = 3) underwent an identical surgical procedure, but without ligation of the aorta. Two weeks after the operation, all animals were euthanized and the hearts were dissected for the isolation of cardiomyocytes or the preparation of total tissue lysates.

### Isolation of Adult Mouse Cardiomyocytes

Each preparation of mouse ventricular cardiomyocytes was performed using the heart from one adult mouse. For experiments involving these cells, three replicates were performed. For each replicate, one cardiomyocytes preparation was used. Adult mouse ventricular cardiomyocytes were isolated by liberase/trypsin digestion following the instructions of the protocol PP00000125 from the Alliance for Cellular Signaling. After isolation, cardiomyocytes were allowed to sediment for 30 min at room temperature and the pellet was re-suspended in 4 ml of preheated and equilibrated plating medium (0.9 × MEM, 5% FCS, 10 mM 2,3-butanedione monoxime, 100 U/ml penicillin and 2 mM L-Glutamine). The shape and density of the cells were checked under the microscope. For immunocytochemistry, cardiomyocytes were plated on laminin-coated cover slides and incubated for 2–4 h at 37°C. For cell fractionations, the cells were seeded into 6-well plates coated with laminin and also incubated for 2–4 h at 37°C.

### Immunocytochemistry and Measurement of Cell Area

The following primary antibodies were used at the indicated dilution: rabbit anti-Ca_v_β_2_ (1:400; Novus Biologicals), mouse anti-α-actinin (1:250; Sigma Aldrich), mouse anti-ryanodine receptor 2 (1:400; Thermo Fischer Scientific). As secondary antibodies, anti-rabbit IgG conjugated to Alexa Fluor 488 and anti-mouse IgG conjugated to Alexa Fluor 633 (Thermo Fischer Scientific) were used. For immunocytochemistry, neonatal rat cardiomyocytes and adult mouse cardiomyocytes were first washed with PBS and then fixed for 10 min with 4% paraformaldehyde. Fixed cells were permeabilized with PBS supplemented with 0.2% Triton-X100 for 15 min at room temperature and blocked with 5% normal goat serum (NGS) (Sigma Aldrich) for 1 h. Cells were incubated overnight at 4°C with the corresponding primary antibodies diluted in 1% NGS. The next day, cells were stained for 2 h at room temperature with the appropriate secondary antibody diluted in 1% NGS. After washing the cells with PBS, coverslips were mounted on glass slides using DAPI mounting medium (Dianova). Confocal fluorescence images of adult mouse cardiomyocytes were acquired on a Leica inverted confocal microscope using a 63 × oil immersion objective. To detect Alexa 488 fluorescence, cells were excited with a 488 nm argon-laser and the emission was monitored at 490–515 nm. The Alexa 633 fluorescence was detected using a 633 nm laser and the emission was monitored at 640–700 nm.

To measure NRC area, cells were α-actinin stained and images were acquired using an Olympus inverted fluorescence microscope. The sizes of 150–200 individual cells per treatment from 20 randomly chosen fields and from 3 replicated experiments were measured using the Image J software. The average cell size obtained in each experiment was used to calculate the mean cell size from the 3 replicated experiments. Only cells lying completely within the fields were quantified. The fraction of nuclear Ca_v_β_2_ in NRCs was calculated by the Manders colocalization coefficient using the JACoP plugin (20) embedded in the Image J software (NIH), which evaluated the ratio of colocalization between Ca_v_β_2_-staining and DAPI-staining in the nucleus, as described Miranda-Laferte et al. (21).

### Cell Fractionations and Western Blots

Cellular fractionations of adult mouse hearts and adult mouse cardiomyocytes were performed following the manufacturer's instructions of the Subcellular Protein Fractionation Kit for Tissues (Thermo Fisher Scientific) and the Subcellular Protein Fractionation Kit for Cultured Cells (Thermo Fisher Scientific), respectively. Protein lysates from NRCs were obtained from cells plated on 6-well plates. After rinsing with 1 × PBS, proteins were extracted for 10 min on ice by adding 120 μl of ice-cold RIPA buffer (25 mM Tris, 150 mM NaCl, 1% NP40, 1% sodium deoxycholate and 0.1% SDS; Thermo Fischer Scientific) supplemented with 1 × Halt protease inhibitor cocktail (Thermo Fisher Scientific). Cells were then scraped off and supernatants were collected after centrifugation at 16,000 × g for 15 min at 4°C. The protein concentrations of the cellular fractions and total protein lysates were measured using the Pierce^TM^ BCA Protein Assay Kit (Thermo Fisher Scientific). For western blot analyses, 50 μg of protein lysates or cellular fractions were resolved on SDS-PAGE, transferred to nitrocellulose membranes, blocked with 5% bovine serum albumin diluted in TBST (10 mM Tris, 150 mM NaCl, 0.5% Tween) and incubated overnight at 4°C with the corresponding primary antibodies: Ca_v_β_2_ (1:1,000, Novus Biologicals), GAPDH (1:5,000, Cell Signaling), sodium potassium ATPase (1:5,000, Abcam), histone H3 (1:1,000, Abcam), calpastatin (1:250, Santa Cruz Biotechnology). On the next day, membranes were washed with TBST and incubated for 1 h at room temperature with anti-rabbit IgG or anti-mouse IgG antibodies conjugated to horseradish peroxidase. Finally, membranes were washed with TBST and the blots were developed using the Pierce^TM^ ECL Western Blotting Substrate (Thermo Fisher Scientific).

### Mass Spectrometry (MS) and Relative Protein Quantification

For MS analysis four biological replicates of each condition were used, including condition 1: wild type NRCs; condition 2: NRCs transduced with scrambled shRNA (shRNAsc, control); condition 3: NRCs transduced with shRNA338 and condition 4: NRCs transduced with shRNA892. Total cell lysates (10 μg in each lane) were separated by short gel SDS-PAGE. Afterwards, the protein bands were excised, hashed and destained by three times alternating 10-min treatments with buffer A (10 mM ammonium bicarbonate, pH 8.3) and buffer B [buffer A + 100% acetonitrile from Merck Millipore in a ratio of 50:50 (v/v)]. After the second incubation with Buffer A, samples were treated with 50 μl of 10 mM DTT (AppliChem) for 1 h at 56°C and with 50 μl of 50 mM iodoacetamide (Merck Millipore) for 45 min at room temperature before continuing with the destaining protocol. Finally, gel pieces were dried in a vacuum concentrator (RVC2-25CD plus, Martin Christ Gefriertrocknungsanlagen). Digestion was initiated by adding 8 μl of trypsin solution (0.015 μg/μl, Serva) and was performed overnight. The digestion was stopped, and the peptides were eluted by incubating the gel pieces two times during 15 min with 30 μl of a 1:1 solution containing 100% acetonitrile and 0.1% (v/v) TFA (Merck Millipore) in an ice-cooled ultrasonic bath. Samples were dried in a vacuum concentrator and re-suspended in 20 μl of 0.1% (v/v) trifluoroacetic acid. Afterwards, the peptide concentration was determined by amino acid analysis (AAA) as described (22). According to the AAA, 200 ng per sample were taken for MS analysis.

Nano-HPLC-MS/MS was performed as previously described (23) by means of LC-MS/MS on an UltiMate 3000 RSLCnano system coupled online to an LTQ Orbitrap Elite mass spectrometer (both Thermo Fischer Scientific). For protein identification via database searches, the raw files were analyzed with the Proteom Discoverer software (v. 1.4.1.14) (Thermo Fisher Scientific) using the Mascot search algorithm (version 2.5) (Matrix Science Ltd.) searching against the UniProtKB/Swiss-Prot database using rat taxonomy (released 2017_1,556,196 sequences entries in the whole database). The database search was performed with the following parameters: trypsin (digestion mode specific); missed cleavages: 2; mass tolerance 5 ppm for precursor and 0.4 Da for fragment ions; modifications: methionine oxidation as dynamic and cysteine carbamidomethylation as fixed; FDR calculation was performed using target decoy PSM validator implemented in the Proteom Discoverer software and FDR threshold was set to <1%.

Label-free quantification was performed by using the Progenesis QI software (Nonlinear Dynamics Ltd.). Raw files generated by the mass spectrometer were imported in the software and all runs were matched to the most suitable run among them (by automatic selection). Afterwards, the software generated a list of features including the m/z values of all measured peptides at a given retention time. The following filters were used at feature level: allowed charge state in the range 2^+^ and 5^+^, reject the features with two or less isotopes. The raw abundances of each feature were automatically normalized in order to correct experimental variations. Experimental setup was set to within subject comparison three groups. Quantified features were then matched to peptide and protein identification by importing the search results generated by proteome discoverer (see protein identification). For quantification, only proteins with at least two peptide counts for identification were used. Additionally, only unique peptides were used for quantification. An ANOVA test to compare the results from condition 2, condition 3, and condition 4 was carried out for statistical evaluation, taking mean differences, the variance and the sample size into account. To exclude regulation depending on transduced shRNA furthermore an additional comparison between wild type NRCs (condition 1) and shRNAsc (condition 2) NRCs was included. Differential proteins from this comparison were not considered as significant regulated proteins.

The mass spectrometry proteomics data have been deposited to the ProteomeXchange Consortium via the PRIDE (REF: PMID: 26527722) partner repository with the dataset identifier PXD016483 and 10.6019/PXD016483.

### Statistical Analyses

All data are presented as the mean ± SEM. The normal distribution and the homoscedasticity of the data were tested using the D'Agostino's-Pearson normality test and the Bartlett's test, respectively. Statistical analyses were performed using the two-tailed unpaired Student's *t*-test to analyze significant differences between two groups or by two-way ANOVA with the Holm-Sidak's method to detect significant differences when variables were dependent of two factors. Data were analyzed using Microsoft Excel, ImageJ (NIH) and software GraphPad Prism version 7 (GraphPad Software Inc.).

## Results

### Ca_v_β_2_ Silencing Enhances Hypertrophy in NRCs

**Figure 1:**
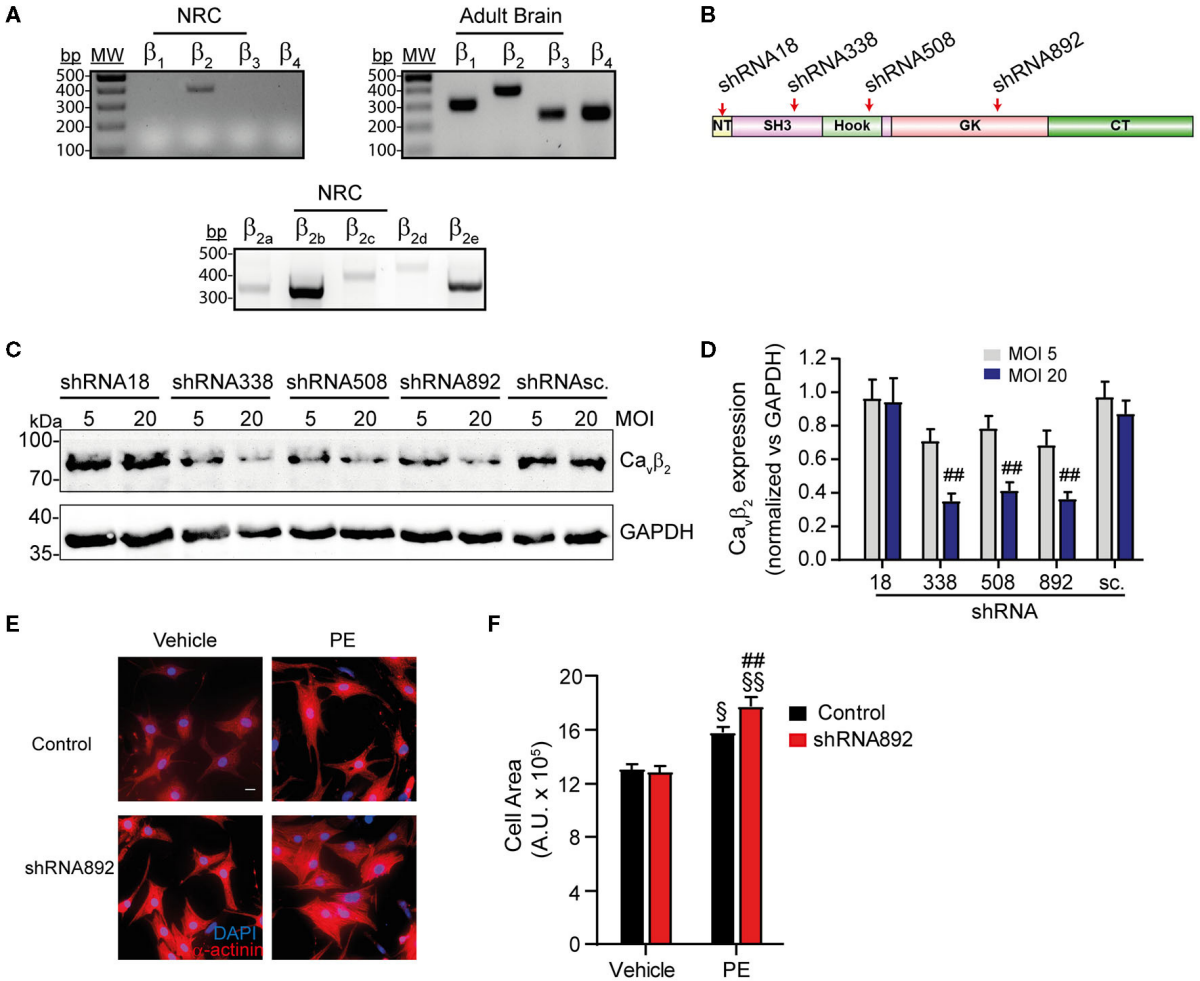
Ca_v_β_2_ downregulation enhances PE-induced hypertrophy of NRCs. **(A)** RT-PCR analysis of the expression of the four Ca_v_β isoforms in NRCs and adult brain. The lower panel shows the expression of the different Ca_v_β_2_ splice variants in NRCs. **(B)** Scheme of the domain organization of Ca_v_β_2_, showing the regions where the designed shRNAs bind: NT (N-terminal), SH3 (Src-3 homology domain), GK (guanylate kinase domain), CT (C-terminal). **(C)** Representative western blot analysis of Ca_v_β_2_ expression in homogenates from NRCs transduced with the different shRNAs for Ca_v_β_2_-silencing and the scrambled shRNA (shRNAsc) used as negative control. Cells were transduced with each shRNA at an adenoviral MOI of 5 or 20. Per lane, 50 μg of protein were loaded. An anti-GAPDH western blot was used as loading control. **(D)** Bar plot of Ca_v_β_2_ expression normalized vs. GAPDH in homogenates from NRCs transduced with the different shRNAs for Ca_v_β_2_-silencing and the scrambled shRNA (shRNAsc.) at an adenoviral MOI of 5 or 20. Mean ± SEM from 3 replicated experiments; ^##^*p* < 0.05 vs. shRNAsc treated at the same MOI (two-way ANOVA with Holm–Sidak's method). **(E)** Representative fluorescent images of control and Ca_v_β_2_-downregulated NRCs (shRNA892) treated with vehicle or 50 μM phenylephrine (PE). Red, α-actinin; Blue, nuclear staining. Scale bar represents 15 μm. **(F)** Bar plot of the mean values of the cell area of control and Ca_v_β_2_-downregulated NRCs (shRNA892) after treatments with vehicle or PE. Mean ± SEM; 150–200 cells per group from 20 randomly chosen fields from 4 replicated experiments were measured; ^§§^*p* < 0.01 vs. vehicle-treated cells in the same group; ^§^*p* < 0.05 vs. vehicle-treated cells in the same group; ^*##*^*p* < 0.05 vs. control treated similarly (two-way ANOVA with Holm–Sidak's method).

RT-PCR analyses demonstrated that of the four Ca_v_β isoforms (Ca_v_β_1_−_4_), Ca_v_β_2_ is the only variant expressed in NRCs (Figure 1A, left panel), while in brain all the isoforms are expressed (Figure 1A, right panel). Of the five different Ca_v_β_2_ splice variants, Ca_v_β_2b_ is the predominantly expressed in NRCs (Figure 1A, lower panel). To investigate the role of Ca_v_β_2_ in cardiomyocyte hypertrophy, we first examined the impact of Ca_v_β_2_ downregulation in NRCs. Four different shRNAs, targeting different regions within the open reading frame of the rat CACNB2 gene (Figure 1B), and a scrambled shRNA (shRNAsc), used as negative control, were designed and delivered into NRCs by adenoviral transduction. All the designed Ca_v_β_2_ shRNA (except shRNA18) target exons in the Src-homology 3-, Hook- and guanylate kinase-domains of the Ca_v_β_2_ gene (Figure 1B). These domains are shared by all the Ca_v_β_2_ splice variants, therefore, all our shRNA (except shRNA18) are expected to downregulate all the Ca_v_β_2_ splice variants. The efficacy of shRNA-mediated knockdown of Ca_v_β_2_ expression was evaluated by western blot 72 h post-adenovirus infection. At a multiplicity of infection (MOI) of 20, the shRNA338 and shRNA892 evoked the strongest Ca_v_β_2_ downregulation (65%) as compared to the control (shRNAsc) (Figures 1C,D).

Ca_v_β_2_ expression in NRCs was downregulated using the previously evaluated shRNA892 and cell hypertrophy was induced by treating the cells with phenylephrine (PE), an α1-adrenergic receptor agonist. As expected, PE treatment significantly increased the size of control cells expressing the shRNAsc, as compared to vehicle-treated cells (Figures 1E,F). However, in cells where Ca_v_β_2_ expression was downregulated this effect was enhanced (Figures 1E,F), indicating that Ca_v_β_2_ plays an inhibitory role in cardiomyocyte hypertrophy.

**Figure 2:**
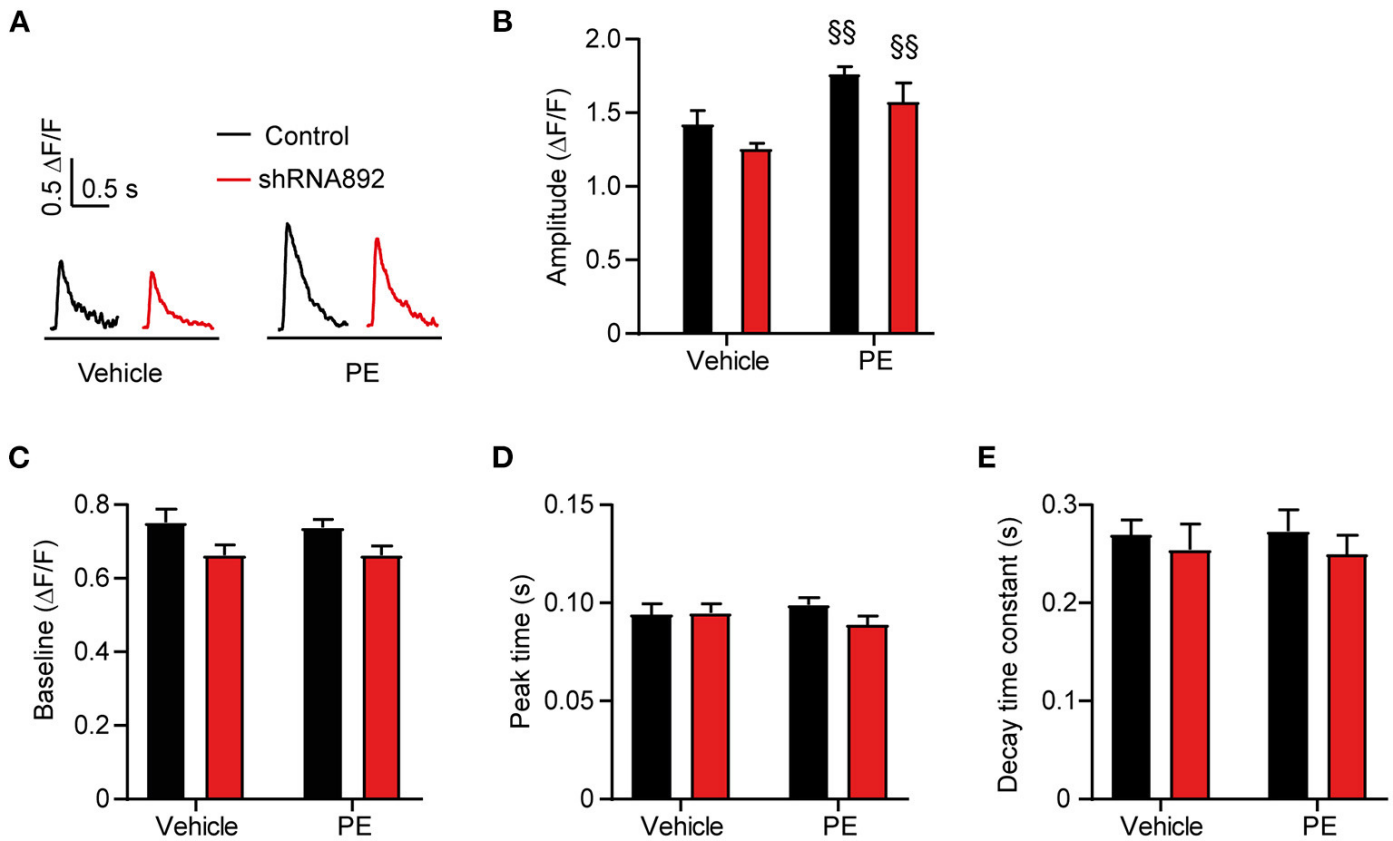
Analyses of calcium transients in control and Ca_v_β_2_-downregulated NRCs after vehicle or PE treatments. **(A)** Representative calcium transients of control (shRNAsc) and Ca_v_β_2_-downregulated NRCs (shRNA892) treated with vehicle or 50 μM phenylephrine (PE) and stimulated at 1.0 Hz. **(B–E)** Bar plots of the mean values of the effect of vehicle or PE treatments on the diastolic calcium, amplitude, peak time, and decay time constant of the calcium transients in control or Ca_v_β_2_-downregulated NRCs. Mean ± SEM; *n* = 11–14 cells per group from 3 different NRC isolations; ^§§^*p* < 0.05 vs. vehicle-treated cells in the same group (two-way ANOVA with Holm–Sidak's method).

Changes in calcium homeostasis leading to the activation of Ca^2+^/calmodulin-dependent kinase II (CAMKII) and calcineurin-dependent signaling pathways have been proposed to play a key role in the development of cardiac hypertrophy (24). Therefore, we investigated whether the inhibitory effect of Ca_v_β_2_ on cardiomyocyte hypertrophy is linked to changes in intracellular calcium levels. Fluorometric Ca^2+^ transient measurements showed an increase in the amplitudes of the Ca^2+^ transients in PE-treated as compared to vehicle-treated NRCs (Figures 2A,B). However, these responses did not differ between the control and Ca_v_β_2_-downregulated cells (Figures 2A,B). Moreover, we also did not observe significant changes in diastolic calcium levels, neither in the kinetics of the Ca^2+^ transients after vehicle or PE treatments in control and Ca_v_β_2_-downregulated (Figures 2C–E). This indicates that the regulatory role of Ca_v_β_2_ in cardiomyocyte hypertrophy does not involve changes in intracellular calcium homeostasis.

### Nuclear Ca_v_β_2_ Expression Decreases After Induction of Cardiomyocyte Hypertrophy *in vitro* and *in vivo*

**Figure 3:**
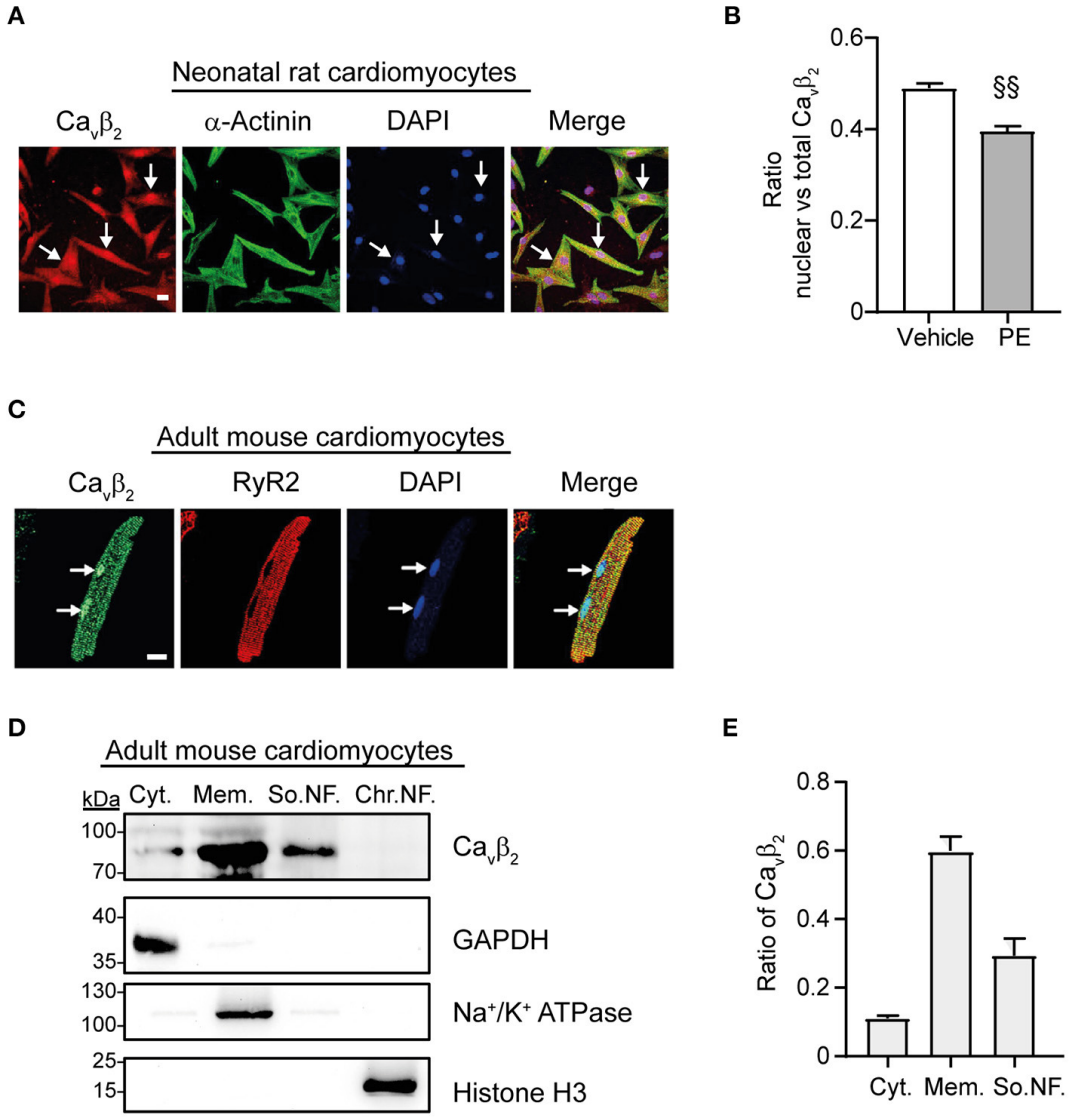
A fraction of Ca_v_β_2_ locates in the nucleus in neonatal and adult cardiomyocytes. **(A)** Confocal fluorescence images of representative NRCs showing a fraction of Ca_v_β_2_ located in the nucleus (indicated with white arrows). Cells were fixed and stained for Ca_v_β_2_ (red), α-actinin (green) and nucleus (DAPI, blue). Scale bar represents 15 μm. **(B)** Bar plot of the Manders coefficient colocalization analyses between Ca_v_β_2_ and DAPI nuclear staining in NRCs after treatment with vehicle or 50 μM phenylephrine (PE). Mean ± SEM; 150–200 cells from 20 randomly chosen fields and from 3 replicated experiments were measured; ^§§^*p* < 0.01 (two-tailed unpaired *t*-test). **(C)** Confocal fluorescence images of a representative adult mouse cardiomyocyte showing a fraction of Ca_v_β_2_ located in the nucleus (indicated with white arrows). Cells were stained for Ca_v_β_2_ (green), ryanodine receptor 2 (RyR2, red) and nucleus (DAPI, blue). Scale bar represents 15 μm. **(D)** Western blots of subcellular fractions from adult mouse cardiomyocytes. Cytosolic fraction (Cyt.), membrane fraction (Mem.), soluble nuclear fraction (So.NF.) and chromatin bound nuclear fraction (Chr.NF.). Per lane, 50 μg of total proteins were loaded. Anti-Ca_v_β_2_ immunoblots detected Ca_v_β_2_ in the nuclear fraction. Immunoblots anti-GAPDH, anti-Na^+^/K^+^ ATPase and anti-histone H3 were used to confirm the presence of cytosolic, membrane, and nuclear proteins in each fraction, respectively. **(E)** Bar plot of the ratio of Ca_v_β_2_ in the different cellular fractions. Mean ± SEM from 3 replicated experiments.

**Figure 4:**
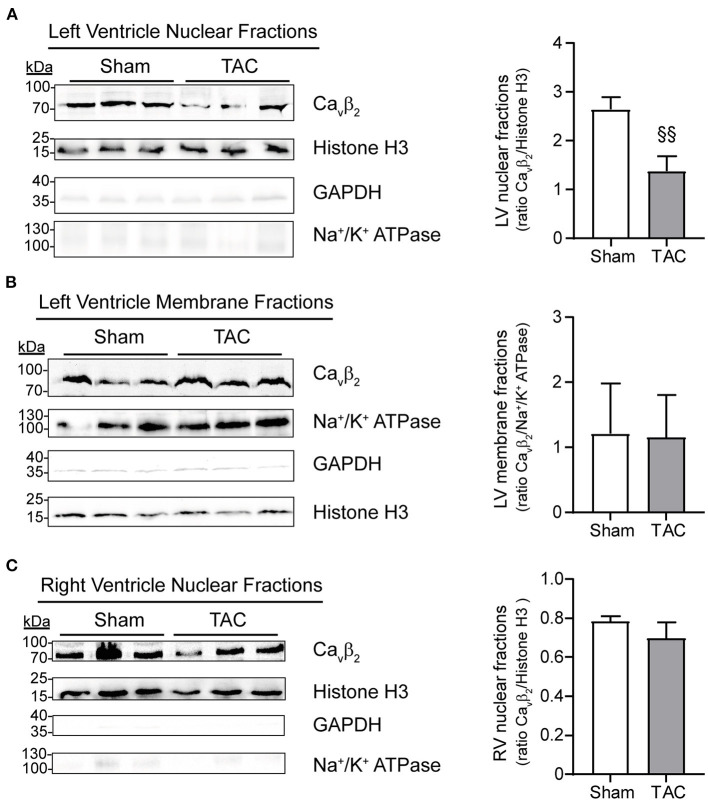
*In vivo* induction of left ventriclulare hypertrophy produces a decrease in nucleus-targeted Ca_v_β_2_. Western blot analyses (left panels) and bar plots of mean values of densitometry analyses from western blots (right panels) of: **(A)** left ventricle (LV) nuclear fractions, **(B)** LV membrane fractions and **(C)** right ventricle (RV) nuclear fractions. Per well, 50 μg of total proteins from sham-operated (*N* = 3) or TAC-operated (*N* = 3) mice were loaded and detected using an anti-Ca_v_β_2_ antibody. Immunoblots to confirm the presence of the compartment-specific protein markers histone H3 (nucleus), Na^+^/K^+^ ATPase (membrane) and GAPDH (cytosol) were also performed. Ca_v_β_2_ expression was normalized to histone-H3 (in **A** and **C**) or to Na^+^/K^+^ ATPase expression (in B). ^§§^*p* < 0.05 vs. sham (two-tailed unpaired *t*-test).

Immunocytochemistry and immunoblot studies after cell fractionation revealed the presence of Ca_v_β_2_ in the nucleus of primary cultured NRCs (Figure 3A). Furthermore, PE-induced hypertrophy of NRCs provoked a significant decrease in the fraction of nuclear Ca_v_β_2_, as compared to vehicle-treated cells (Figure 3B). In adult mouse cardiomyocytes, most Ca_v_β_2_ molecules are in proximity to the ryanodine receptors (Figure 3C). This pool belongs to the LTCC complexes that are targeted to the t-tubules and are located at ~10–20 nm from the sarcoplasmic reticulum membrane (25). However, immunocytochemical and cell fractionation analyses also showed a pool of nuclear Ca_v_β_2_ in adult cardiomyocytes (Figures 3C–E). To assess whether changes in nuclear Ca_v_β_2_ expression also accompany cardiac hypertrophy *in vivo*, we performed surgical transverse aortic constrictions (TAC) in mice. As expected, 2 weeks after TAC mice exhibited a significant ~20% increase in cardiomyocyte size as compared to sham-operated animals (Supplementary Figures 1A,B). Moreover, the heart-to-body weight (Supplementary Figure 1C) and the left ventricle-to-body weight (Supplementary Figure 1D) ratios were increased after TAC, without changes in the right ventricle-to-body weight ratio (Supplementary Figure 1E). Notably, a marked decrease in the expression of nuclear Ca_v_β_2_ in the left ventricle was associated with the development of cardiac hypertrophy after TAC (Figure 4A), while the levels of membrane-associated Ca_v_β_2_ in the left ventricle and of nuclear Ca_v_β_2_ in the right ventricle were unaltered (Figures 4B,C). These results suggest that nucleus-targeted Ca_v_β_2_ could play a role as regulator of cardiomyocyte hypertrophy.

### Ca_v_β_2_ Nuclear Overexpression Abolishes *in vitro* PE-Induced Cardiomyocyte Hypertrophy

**Figure 5:**
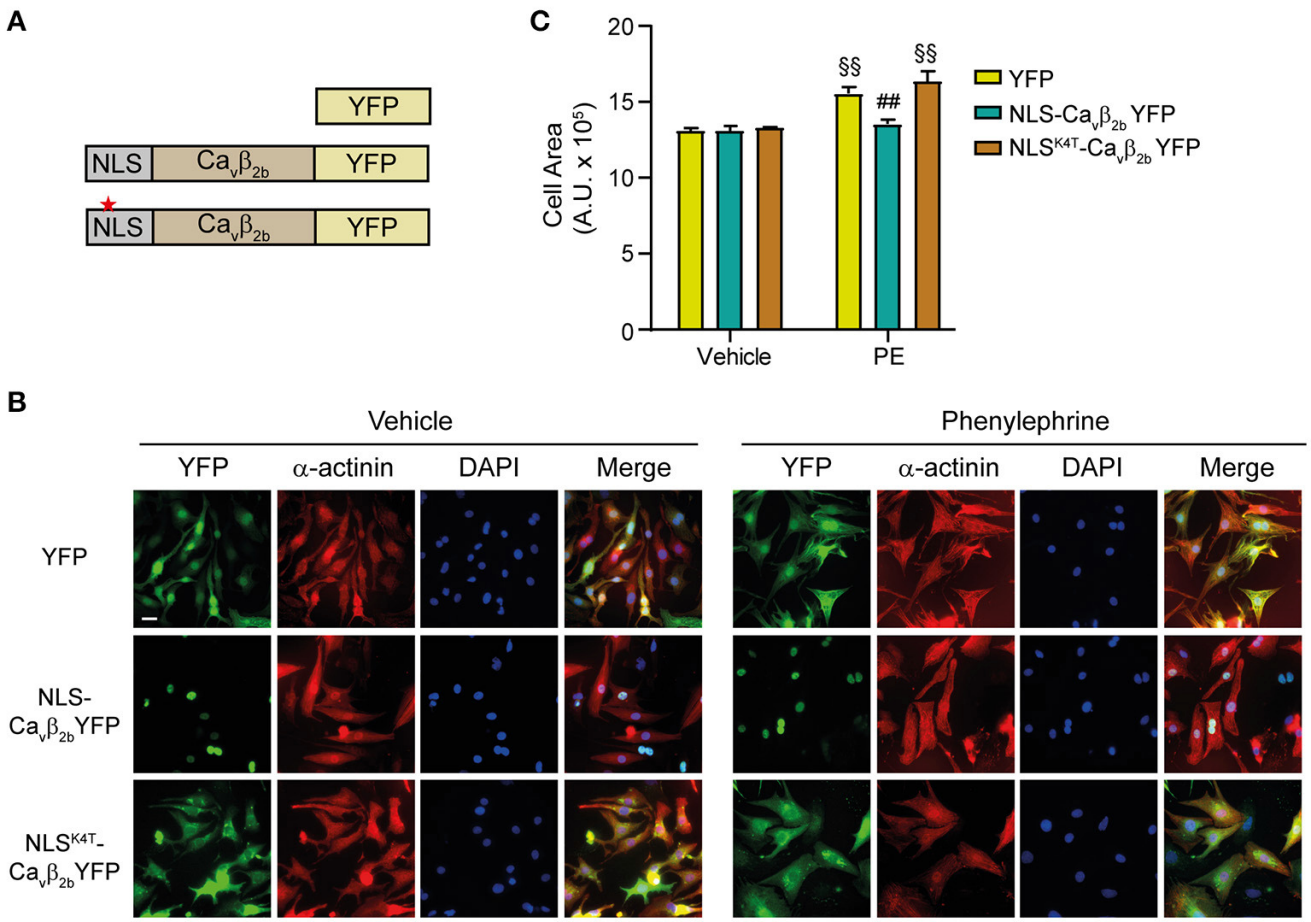
Nuclear overexpression of Ca_v_β_2_ abolishes *in vitro* PE-induced cardiomyocyte hypertrophy. **(A)** Schematic representations of the three evaluated constructs. To achieve a complete Ca_v_β_2_ nuclear-targeting, the nuclear localization signal (NLS) of the SV40 large T-antigen (PPKKKRKV) was inserted at the N-terminus of the Ca_v_β_2b_-YFP construct (NLS-Ca_v_β_2b_-YFP). YFP and a construct with a mutated NLS (NLS^K4T^-Ca_v_β_2b_-YFP, red star) were used as negative controls. **(B)** Fluorescent images of NRCs expressing the controls (YFP or NLS^K4T^-Ca_v_β_2b_-YFP) or nuclear overexpressed Ca_v_β_2b_ (NLS-Ca_v_β_2b_-YFP) and treated with vehicle or 50 μM phenylephrine (PE). Cells were stained for α-actinin (red) and nucleus (DAPI, blue), while the YFP fluorescence was directly monitored. Scale bar represents 25 μm. **(C)** Bar plot of the cell area analyses of NRCs expressing YFP, NLS-Ca_v_β_2b_-YFP or NLS^K4T^-Ca_v_β_2b_-YFP after the treatment with vehicle or PE. Mean ± SEM; 150–200 cells per group from 20 randomly chosen fields from 3 replicated experiments were measured; ^§§^*p* < 0.01 vs. vehicle-treated cells in the same group; ^*##*^*p* < 0.05 vs. YFP expressing cells treated similarly (two-way ANOVA with Holm–Sidak's method).

As previously described, Ca_v_β_2b_ is the predominantly expressed variant in NRCs (Figure 1A, lower panel). Therefore, to investigate the role of nucleus-targeted Ca_v_β_2_ in cardiomyocyte hypertrophy, we designed a plasmid where the nuclear localization signal (NLS) of the SV40 T antigen was inserted at the N-terminus of a Ca_v_β_2b_-YFP fusion protein (NLS-Ca_v_β_2b_-YFP) (Figure 5A). Two constructs encoding the expression of YFP or of an inactive NLS signal at the N-terminus of the Ca_v_β_2b_-YFP (NLS^K4T^-Ca_v_β_2b_-YFP) were used as negative controls (Figure 5A). The three constructs were cloned into adenoviral vectors and transduced into NRCs. As expected for a relatively low molecular weight protein that can diffuse passively through the nuclear pores, YFP was equally distributed through the cytosol and the nucleus (Figure 5B). Insertion of an NLS into the Ca_v_β_2b_ open reading frame, mediated the complete targeting of the protein to the nucleus (Figure 5B), an effect that was prevented in most of the Ca_v_β_2_-transduced cells by the insertion of an inactive NLS (NLS^K4T^-Ca_v_β_2b_ -YFP) (Figure 5B). We next assessed the impact of nuclear Ca_v_β_2_ overexpression on agonist-induced hypertrophy in NRCs. PE-treatment induced a significant increase in the size of YFP-transduced cells (Figures 5B,C). Similar responses were observed in NRCs expressing the non-nucleus-targeted NLS^K4T^-Ca_v_β_2b_-YFP protein (Figures 5B,C). However, in NRCs overexpressing a nucleus-targeted Ca_v_β_2b_ (NLS-Ca_v_β_2b_-YFP), PE-induced hypertrophy was completely abolished (Figures 5B,C). These results indicate a regulatory role of the nucleus-targeted Ca_v_β_2b_ in cardiomyocyte hypertrophy.

### Ca_v_β_2_ Regulates the Expression of Cardiomyocyte Proteins

**Figure 6:**
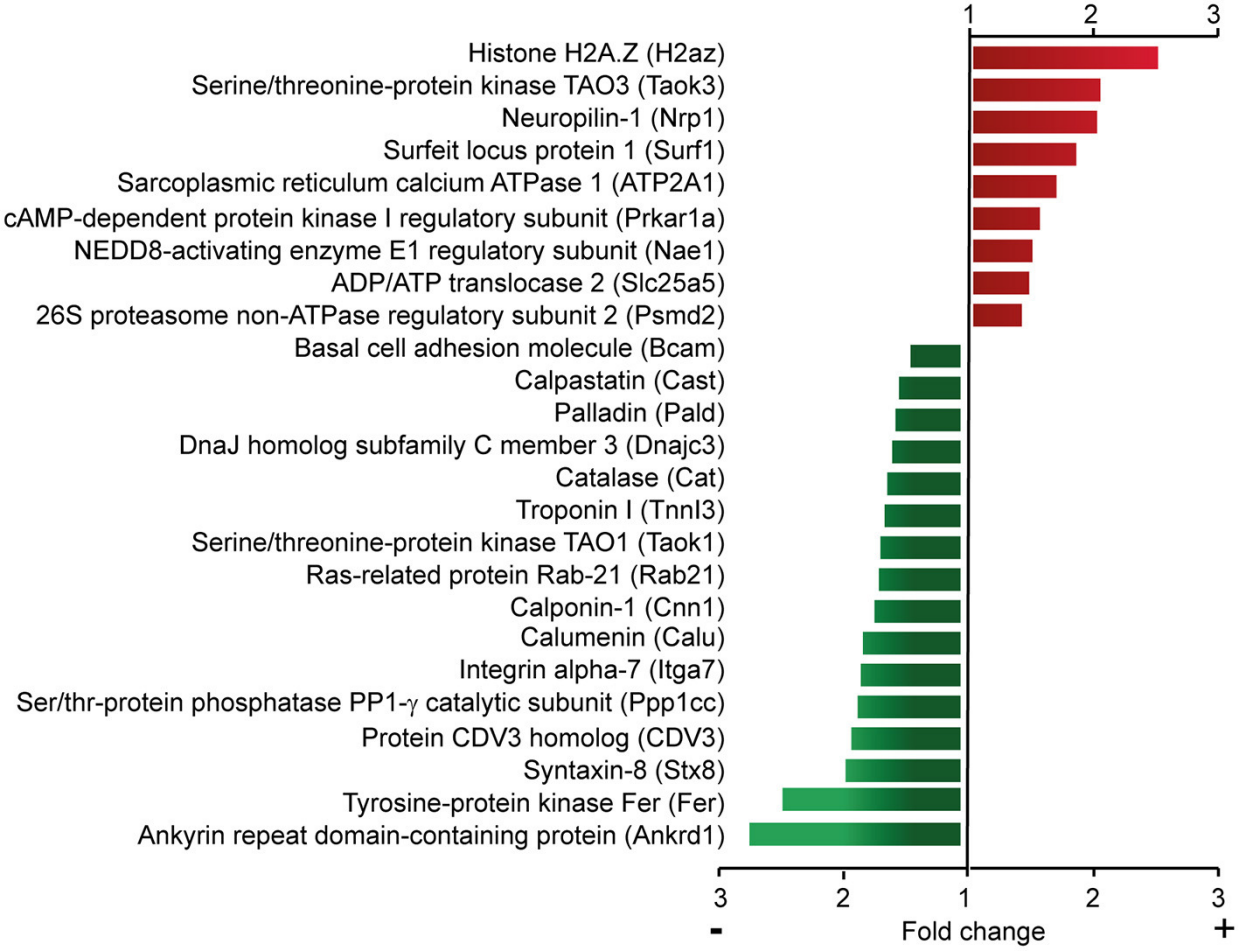
Quantitative proteomic analysis of control and Ca_v_β_2_-downregulated NRCs. Subset of upregulated (red) or downregulated (green) proteins identified by quantitative mass-spectrometry-based analyses after Ca_v_β_2_ downregulation in NRCs. A complete list of proteins is provided in Supplementary Table 1.

As described above, changes in calcium homeostasis do not mediate the regulatory role of Ca_v_β_2_ in cardiomyocyte hypertrophy. Therefore, we performed quantitative mass-spectrometry-based analyses to get mechanistic insights into how Ca_v_β_2_ knockdown influences the expression of cardiomyocyte proteins involved in hypertrophy. Quantitative comparison of the proteome of wild type, shRNAsc-(control), shRNA338- and shRNA892-transduced NRCs resulted in 1,471 quantified proteins with at least two peptide counts for protein identification and revealed that Ca_v_β_2_ silencing significantly (ANOVA *p* < 0.05) upregulated the expression of 16 proteins and decreased the expression of 28 proteins (Supplementary Table 1). A subset of these proteins is shown in Figure 6. The downregulated proteins included the ankyrin repeat domain-containing protein 1, Ras-related protein Ras-21, calpastatin, calponin, troponin I, and the catalytic subunit of serine/threonine phosphatase PP-1. Some of the upregulated proteins are the regulatory subunit of the cAMP-dependent protein kinase, neuropilin, basal cell adhesion molecule and serine-threonine-protein kinase TAO3. These results suggest that Ca_v_β_2_ could be involved in the regulation of a wide set of genes controlling cardiomyocyte development, calcium homeostasis, muscular contraction and cellular metabolism. Moreover, some of these genes, including the ones coding for catalase (26) and calpastatin (15), have been involved in the development of cardiac hypertrophy and could therefore provide the link between Ca_v_β_2_ expression and this pathology.

### Ca_v_β_2_ Regulates Calpastatin Expression and Calpain Activity in Cardiomyocytes

**Figure 7:**
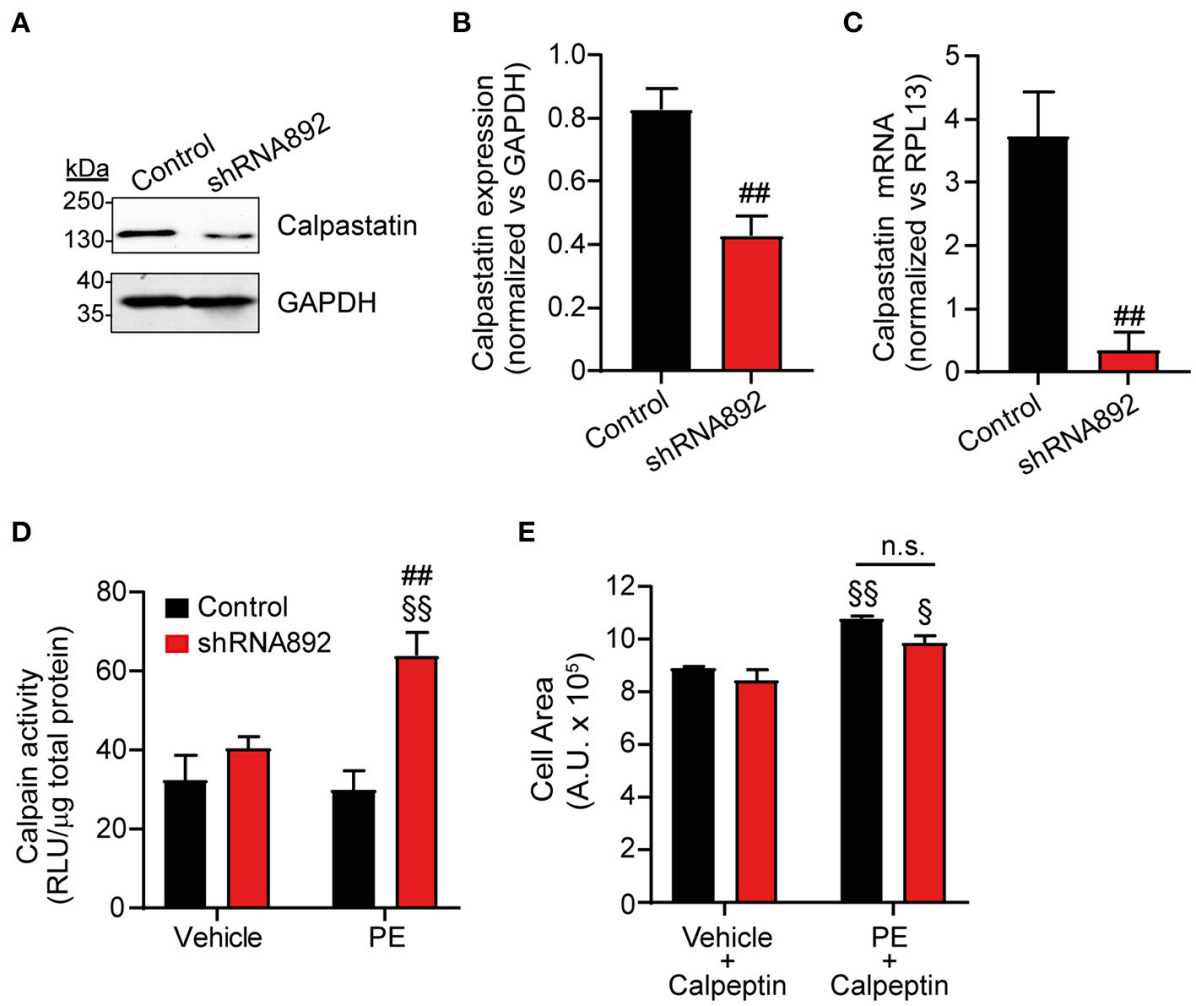
Inhibition of calpain activity abolishes the enhanced PE-induced cardiomyocyte hypertrophy in Ca_v_β_2_-downregulated NRCs. **(A)** Representative western blot analysis of calpastatin expression in homogenates from control and Ca_v_β_2_ downregulated NRCs. Per well, 50 μg of protein were loaded. Anti-GAPDH western blot was used as loading control. **(B)** Bar plot of calpastatin expression normalized vs. GAPDH in homogenates from control and Ca_v_β_2_ downregulated NRCs. Mean ± SEM of 3 different experiments; ^*##*^*p* < 0.05 (two-tailed unpaired *t*-test). **(C)** Bar plot of the qRT-PCR analyses of calpastatin expression in control and Ca_v_β_2_ downregulated NRCs; ^*##*^*p* < 0.05 (two-tailed unpaired *t*-test). **(D)** Bar plot of calpain activity analyses of control and Ca_v_β_2_-downregulated NRCs after the treatment with vehicle or 50 μM phenylephrine (PE). Mean ± SEM of 6 different experiments; ^§§^*p* < 0.05 vs. vehicle-treated cells in the same group; ^*##*^*p* < 0.01 vs. control cells treated similarly (two-way ANOVA with Holm–Sidak's method). **(E)** Bar plot of the mean values of the cell area of the control and Ca_v_β_2_-downregulated NRCs after vehicle and 25 μM of calpeptin or PE and calpeptin treatments. Mean ± SEM; 150–200 cells per group from 20 randomly chosen fields from 3 replicated experiments were measured; ^§§^*p* < 0.01 vs. vehicle and calpeptin-treated cells in the same group; ^§^*p* < 0.05 vs. vehicle and calpeptin-treated cells in the same group; n.s. non-significant (two-way ANOVA with Holm–Sidak's method).

Calpains are calcium-dependent non-lysosomal cysteine proteases consisting of a calpain catalytic subunit, a small regulatory subunit and calpastatin, which is an endogenous calpain-specific inhibitor. Increased calpain activity has been associated with different cardiac diseases including heart hypertrophy (14, 15). As mentioned above, proteomic analyses detected a significant decrease in calpastatin expression in Ca_v_β_2_-downregulated cells (Figure 6 and Supplementary Table 1). RT-PCR and western blot analyses confirmed the decrease in calpastatin expression at the mRNA and protein levels, respectively, in Ca_v_β_2_-downregulated NRCs as compared to controls (Figures 7A–C). Hence, we hypothesized that reduced calpastatin expression leads to augmented calpain activity thereby contributing to the enhanced hypertrophic response to PE observed in Ca_v_β_2_-downregulated NRCs. Under baseline, vehicle conditions, calpain activity did not differ between controls and Ca_v_β_2_-downregulated cells (Figure 7D). In addition, after PE treatments control cells did not show any changes in calpain activity (Figure 7D). However, in Ca_v_β_2_-downregulated cells, PE treatments induced a 2-fold increase of calpain activity as compared to vehicle treatments (Figure 7D). To evaluate if this increase in calpain activity observed in Ca_v_β_2_-downregulated cells could be related to their enhanced hypertrophic response observed after PE treatments, we decide to analyze the effect of inhibiting calpain activity on cardiomyocyte hypertrophy. To this aim, we treated controls and Ca_v_β_2_-downregulated cells with calpeptin, a potent calpain inhibitor. After calpeptin treatments, controls and Ca_v_β_2_-downregulated cells displayed similar cell areas, but they were smaller as compared to vehicle treated cells (Figure 7E). Moreover, when PE-induced hypertrophy was evaluated in the presence of calpeptin, the enhanced hypertrophic response of Ca_v_β_2_-downregulated cells was abolished and the differences in cell area between control and Ca_v_β_2_-downregulated cells were not observed. These results indicate that in Ca_v_β_2_-downregulated cells an increase in calpain activity contributes to enhanced hypertrophy.

## Discussion

Most studies about the function of Ca_v_β have focused on its possible regulatory effects on LTCCs trafficking and activity. However, recent studies have challenged the classical view of Ca_v_β as an LTCC regulator, by demonstrating that Ca_v_β-free LTCC complexes have a normal activity in cardiomyocytes (2, 10) and that in other cell types Ca_v_β can participate in non-LTCC-related cellular processes (11–13). The possible role of Ca_v_β_2_ in the development of cardiac hypertrophy has been explored using *in vitro* and *in vivo* models (27, 28). However, one of these reports just demonstrated a 23% reduction in Ca_v_β_2_ expression after Ca_v_β_2_ silencing (27). The other studied (28) used a transgenic mouse model overexpressing a Ca_v_β_2_ splice variant (Ca_v_β_2a_), that is not detected in the cardiomyocytes and that is normally palmitoylated at the plasma membrane (29, 30), which could alter its translocation to other cellular compartments or its interaction with non LTCC-related proteins after its overexpression in cardiomyocytes.

To further characterize the possible functions of Ca_v_β_2_ in cardiomyocyte hypertrophy, we used NRCs as model system. Our study shows that Ca_v_β_2_ downregulation enhances α1-adrenergic receptor-mediated hypertrophy through a calpain-mediated signaling pathway. Moreover, Ca_v_β_2_-downregulated NRCs displayed normal calcium transients after electrical stimulations at 1 Hz, confirming that, as previously suggested (2, 10), an association between the Ca_v_α_1_ channel pore-forming subunit and Ca_v_β_2_ is neither necessary for the membrane localization and regulation of the LTCC channels, nor for the activation of the calcium-induced calcium release mechanism in cardiomyocytes at low stimulation frequencies. It has been reported that adult mice with a conditional cardiomyocyte-specific deletion of the Ca_v_β_2_ gene do not display cardiac hypertrophy or dysfunction under resting physiological conditions (2). Consistently, we showed in the present study that Ca_v_β_2_-downregulated NRCs display normal cell areas and calcium transients under baseline conditions, but undergo enhanced hypertrophy in response to PE stimulations. This observation suggests that Ca_v_β_2_ does not participate in normal physiological myocyte growth, but it moderates the pathological growth in response to hypertrophic stimuli like PE.

We assessed the intracellular localization of Ca_v_β_2_. As expected, in adult mouse cardiomyocytes most of Ca_v_β_2_ is integrated into the LTCC complexes located at the t-tubules that are in proximity to the ryanodine receptors in the sarcoplasmic reticulum (Figure 3C). However, we also provide the first evidence that in primary isolated adult mouse and neonatal rat cardiomyocytes, a pool of Ca_v_β_2_ is located in the nucleus as described for the cardiac HL-1 cell line (31).

It has been reported that Ca_v_β_2_ regulates the expression of diverse genes (32–34). Therefore, in cardiomyocytes, the pool of Ca_v_β_2_ detected in the nucleus could have a transcriptional role or regulate the function of transcription factors and, in consequence, gene expression, as previously described in neurons and skeletal muscle cells (32–34). Additionally, as shown in the present study, *in vitro* and *in vivo* induction of cardiomyocyte hypertrophy promotes a decrease in the nuclear Ca_v_β_2_ fraction, without changes at the RNA level in the expression of the Ca_v_β_2_ splice variants (Supplementary Figure 2). This result suggests that changes in the dynamics of the cytosolic-nuclear trafficking of Ca_v_β_2_ occur during this pathophysiological process.

The mechanism of Ca_v_β_2_ trafficking to the nucleus in primary cardiomyocytes is still unclear. The relatively high molecular weight of Ca_v_β_2_ (72 kDa) is far beyond the size allowing passive diffusion through the nuclear pores (35). Therefore, the presence of a nuclear localization signal within the amino acid sequence of Ca_v_β_2_ or its interaction with a nucleus-targeted protein should be necessary for Ca_v_β_2_ nuclear translocation. Our *in silico* predictions support the latter suggestion, since they failed to identify a predicted NLS within the sequence of Ca_v_β_2_.

An upregulation in the rate of Ca_v_β_2_ nuclear export could also explain the downregulation of nucleus-targeted Ca_v_β_2_ during cardiac hypertrophy. However, as for nuclear import, the mechanism of Ca_v_β_2_ nuclear export still needs to be clarified. An increase in histone deacetylase-5 (HDAC5) nuclear export, mediated by the protein kinase C (PKC)-dependent phosphorylation of HDAC5, has been shown to promote cardiac hypertrophy (36). Moreover, *in vitro* and *in vivo* induction of cardiac hypertrophy by PE-treatments or TAC, respectively, leads to PKC activation (37). Therefore, since Ca_v_β_2_ has been reported to be phosphorylated by PKC (38, 39), PKC-mediated phosphorylation of Ca_v_β_2_ after PE-treatments or TAC could also increase Ca_v_β_2_ nuclear export and upregulate cardiomyocyte hypertrophy. This is an exciting hypothesis that could be tested in the future.

Ca_v_β has been reported to interact with members of the RGK protein family (Ras-related small GTP-binding proteins) (40–42) and in the cardiomyocyte HL-1 cell line, these interactions were sufficient to mediate the nuclear targeting of Ca_v_β_2_ (31). A downregulation in the expression of RGK proteins during *in vitro* and *in vivo*-induced cardiac hypertrophy has also been described (43). Consequently, the interaction of Ca_v_β_2_ with members of the RGK protein family could also mediate its nuclear-translocation and explain also the decrease of nucleus-targeted Ca_v_β_2_ during cardiac hypertrophy.

We confirmed the relevance of Ca_v_β_2_ nuclear targeting in cardiomyocyte hypertrophy with the observation that a full nuclear translocation of Ca_v_β_2_, mediated by a viral NLS, abolished PE-induced hypertrophy. Together these results indicate that nuclear Ca_v_β_2_ participates in the regulation of cardiomyocyte hypertrophy.

To assess if changes in Ca_v_β_2_ levels could influence cardiomyocyte protein expression, we performed quantitative proteomic analyses of control and Ca_v_β_2_-downregulated NRCs. Silencing of Ca_v_β_2_ altered the expression of various proteins. Interestingly, despite the upregulation of the SERCA2a expression and the low expression of calcium binding proteins as Calponin-1 or Calumenin, Ca_v_β_2_-downregulated NRCs have similar diastolic calcium levels and equivalent decay time of the calcium transient as control cells. This result indicates that probably these changes observed in these cells are mutually compensated and that calcium homeostasis is not affected after Ca_v_β_2_-downregulation. Moreover, we cannot exclude that SERCA2a overexpression in Ca_v_β_2_-downregulated NRCs could be counteracted by an increase in phospholamban levels or in its dephosphorylation state. This could explain the similar decay time constants observed in the calcium transients from control and Ca_v_β_2_-downregulated NRCs.

Reactive oxygen species (ROS) can mediate hypertrophic signals (44). Our proteomics approach revealed that Ca_v_β_2_-downregulated NRCs have a decrease in catalase expression as compared to controls. This could indicate elevated ROS levels and oxidative stress in these cells, which could contribute to their enhanced hypertrophy (45). Mitochondria are an important source of ROS (46) and it is known that the functional association between LTCC and mitochondria plays an important role in the development of cardiac hypertrophy (47). Moreover, it has been demonstrated that LTCC activity can affect the mitochondrial membrane potential and the mitochondrial activity in a calcium-independent manner. In this mechanism the interaction of Ca_v_β_2_ with actin filaments plays a central role (8, 17, 48). Consequently, it would be very interesting to test the impact of Ca_v_β_2_ downregulation in mitochondrial activity and cardiac hypertrophy.

It is important to note that calpastatin, an endogenous inhibitor of the calcium-dependent protease calpain, was one of the proteins downregulated by Ca_v_β_2_ knockdown. Regardless of their lower calpastatin levels as compared to controls, Ca_v_β_2_-downregulated NRCs have normal calpain activity and cell areas under vehicle conditions. This indicates that just a decrease in calpastatin expression is not enough to induce an increase in calpain activity and that probably a hypertrophic stimulus inducing an increase in intracellular Ca^2+^ concentration is also needed. Accordingly, PE treatments of Ca_v_β_2_-downregulated NRCs, which produced an increase in the amplitude of the Ca^2+^ transients (Figure 2B), also induced an upregulation in calpain activity and an enhanced hypertrophy as compared to control cells. An increased calpain activity has been reported in some pathophysiological processes like cardiac hypertrophy (14, 15). A rise in the intracellular Ca^2+^ concentration, mediated by the activation of G-protein-coupled α1-adrenergic receptors, triggers hypertrophic signaling through calcineurin-induced nuclear factor of activated T-cells (NFAT) activation or CAMKII-mediated histone deacetylase inactivation (24). However, the activation of the calcineurin/NFAT signaling pathway can also be facilitated by calpain-mediated proteolytic cleavage of the calcineurin autoinhibitory domain (14), an irreversible activation mechanism that, given its persistent character, could lead to a stronger hypertrophic phenotype. Therefore, under pathological conditions, calpain-mediated and calpain-independent mechanisms can contribute to cardiac hypertrophy.

Inhibiting calpain activity with calpeptin in NRCs produces a decrease in the cell size of control and Ca_v_β_2_-downregulated NRCs. Nevertheless, calpeptin treatments do not affect PE-induced cardiomyocyte hypertrophy in controls cells, indicating that in those cells PE-induced hypertrophy occurs mainly through the activation of calpain-independent pathways. However, in Ca_v_β_2_-downregulated NRCs, which display high calpain activity and enhanced cardiomyocyte hypertrophy after treatment with PE, the pharmacological inhibition of calpain activity abolished the enhanced PE-induced cardiomyocyte hypertrophy. This suggests that in cardiomyocytes an upregulation of calpain activity probably switches on calpain-dependent hypertrophic pathways. Moreover, the irreversibility and persistent character of the calpain-mediated calcineurin activation could explain the increases in cardiomyocyte hypertrophy observed in Ca_v_β_2_-downregulated NRCs (14).

Our study focused on the role of Ca_v_β_2_ in cardiomyocyte hypertrophy, which is a precursor to heart failure. To this aim, we designed *in vitro* and *in vivo* models, which resemble cardiac hypertrophy but not heart failure. An enhanced calpain activity, which promotes the proteolytic cleavage of diverse cardiac proteins, has been reported during heart failure (15, 49). The calpain-mediated cleavage of cardiac proteins during heart failure seems to affect cardiac homeostasis and to be crucial for the development of this disease (50). Nevertheless, it has been demonstrated that the cleavage of junctophilin-2 by calpain releases an N-terminal peptide, which can translocate to the nucleus and ultimately has a protective effect against heart failure (51). Therefore, the consequences of the increased calpain activity observed during this pathology remain contradictory. Previous studies have shown an upregulation of Ca_v_β_2_ expression in human failing hearts (52–54). Hence, it would be interesting to evaluate under heart failure conditions the effects on calpain activity of Ca_v_β_2_ downregulation and its consequences.

## Data Availability Statement

The datasets presented in this study can be found in online repositories. The names of the repository/repositories and accession number(s) can be found below: http://www.proteomexchange.org/, PXD016483.

## Ethics Statement

The animal study was reviewed and approved by Regierung von Unterfranken.

## Author Contributions

EM-L, PE-N, KM, KB, and MK: designed the research. SP and EM-L: performed the molecular biology experiments. SP, SB, YC-G, and AS: isolated and cultured neonatal rat cardiomyocytes. SP, YC-G, EM-L, and PE-N: prepared the adenoviruses. SP, EM-L, and PE-N: performed the calcium measurements. KB, SP, YC-G, and KP: performed the mass spectrometry analyses. SP, CH, and EM-L: performed the immunocytochemistry and the biochemical experiments. KV: isolated the adult mouse cardiomyocytes. MA: performed the transverse aortic constrictions in mice. SP, KB, and EM-L: analyzed the data. EM-L: wrote the manuscript. All authors contributed to the article and approved the submitted version.

## Conflict of Interest

The authors declare that the research was conducted in the absence of any commercial or financial relationships that could be construed as a potential conflict of interest.
