# Supplementary material for: The β2-Subunit of Voltage-Gated Calcium Channels Regulates Cardiomyocyte Hypertrophy
Source: Front Cardiovasc Med. 2021 Jul 7;8:704657. doi: 10.3389/fcvm.2021.704657 (PMC8292724; doi:10.3389/fcvm.2021.704657)
Supplement: Supplementary file 1 [file Table_1.pdf]

Table S1. List of upregulated and downregulated proteins identified by LC-MS analyses in $Ca_v\beta_2$-downregulated NRCs

Upregulated Proteins

Acc Num	Description	Peptide count	Unique peptides	Anova p-value	Fold Change
P0C0S7	Histone H2A.Z	7	3	0.039372	2.36
Q53UA7	Serine/threonine-protein kinase TAO3	6	1	0.0478008	1.93
Q9QWJ9	Neuropilin-1	2	2	0.0249368	1.81
Q9Z330	DNA (cytosine-5)-methyltransferase 1	2	1	0.0056253	1.71
Q9QXU2	Surfeit locus protein 1	2	2	0.0418875	1.60
Q8K1Q0	Glycylpeptide N-tetradecanoyltransferase 1	5	3	0.0293166	1.59
Q56A27	Nuclear cap-binding protein subunit 1	3	2	0.0413061	1.54
Q64578	Sarcoplasmic/endoplasmic reticulum calcium ATPase 1	27	2	0.0246706	1.40
Q9QZ81	Protein argonaute-2	2	1	0.0310035	1.39
P09456	cAMP-dependent protein kinase type I-alpha regulatory subunit	26	18	0.0075715	1.32
Q9Z1A5	NEDD8-activating enzyme E1 regulatory subunit	4	3	0.023091	1.31
Q09073	ADP/ATP translocase 2	36	7	0.0402633	1.28
P16036	Phosphate carrier protein, mitochondrial	17	15	0.0430052	1.23
Q4FZT9	26S proteasome non-ATPase regulatory subunit 2	17	16	0.0412072	1.17
P14882	Propionyl-CoA carboxylase alpha chain, mitochondrial	20	17	0.0137137	1.12
Q03346	Mitochondrial-processing peptidase subunit beta	12	12	0.0349569	1.11

Downregulated Proteins

Acc Num	Description	Peptide count	Unique peptides	Anova p-value	Fold Change
Q8R560	Ankyrin repeat domain-containing protein 1	6	6	0.0002578	2.99
P09760	Tyrosine-protein kinase Fer	4	1	0.031466	2.62
Q9Z2Q7	Syntaxin-8	2	2	0.0025648	2.04
Q5XIM5	Protein CDV3 homolog	3	3	0.0260633	1.82
P63088	Serine/threonine-protein phosphatase PP1-gamma catalytic subunit	11	1	0.0219534	1.79
P50442	Glycine amidinotransferase, mitochondrial	2	2	0.0024302	1.79
Q63258	Integrin alpha-7	5	5	0.0143161	1.76
O35783	Calumenin	5	5	0.0395409	1.74
P29534	Vascular cell adhesion protein 1	2	1	0.0481784	1.73
O35760	Isopentenyl-diphosphate Delta-isomerase 1	4	4	0.0165448	1.61
Q08290	Calponin-1	3	2	0.0149159	1.56
Q6AXT5	Ras-related protein Rab-21	3	3	0.043885	1.55
O88664	Serine/threonine-protein kinase TAO1	5	1	0.0500006	1.52
P23693	Troponin I, cardiac muscle	15	13	0.0423728	1.50
P04762	Catalase	30	30	0.0393255	1.44
P15800	Laminin subunit beta-2	5	3	0.007082	1.41
Q5PQP9	Armadillo repeat-containing protein 5	2	1	0.0429086	1.41
Q9ESN0	Protein Niban	4	4	0.0441999	1.38
P47198	60S ribosomal protein L22	3	2	0.0238242	1.33
B2GV06	Succinyl-CoA:3-ketoacid coenzyme A transferase 1, mitochondrial	29	26	0.0220293	1.33
Q9R0T3	DnaJ homolog subfamily C member 3	3	3	0.0364996	1.29
Q5U367	Procollagen-lysine,2-oxoglutarate 5-dioxygenase 3	4	4	0.0122586	1.28
P51583	Multifunctional protein ADE2	7	6	0.0040127	1.23
P0C5E3	Palladin (Fragment)	9	9	0.0254724	1.21
P12001	60S ribosomal protein L18	8	4	0.0261498	1.20
P62083	40S ribosomal protein S7	10	9	0.0225552	1.19
P27321	Calpastatin	4	2	0.0469852	1.18
Q9ESS6	Basal cell adhesion molecule	9	9	0.0249939	1.15
