# Supplementary material for: The β2-Subunit of Voltage-Gated Calcium Channels Regulates Cardiomyocyte Hypertrophy
Source: Front Cardiovasc Med. 2021 Jul 7;8:704657. doi: 10.3389/fcvm.2021.704657 (PMC8292724; doi:10.3389/fcvm.2021.704657)
Supplement: Supplementary file 2 [file Data_Sheet_1.pdf]

The β_2-subunit of voltage-gated calcium channels regulates cardiomyocyte hypertrophy

Simone Pickel[1], Yiliam Cruz-Garcia[1], Sandra Bandleon[2], Katalin Barkovits[3,4], Cornelia Heindl[1], Katharina Völker[1], Marco Abeßer[1], Kathy Pfeiffer[3,4], Alice Schaaf[2], Katrin Marcus[3,4], Petra Eder-Negrin[2], Michaela Kuhn[1,2] and Erick Miranda-Laferte[5,1*]

From the. [1]Institute of Physiology, University of Würzburg, Würzburg, Germany; [2]Comprehensive Heart Failure Center, University Hospital Würzburg, Würzburg, Germany; [3]Medizinisches Proteom-Center, Medical Faculty, Ruhr-University Bochum, Bochum, Germany. [4]Medical Proteome Analysis, Center for Proteindiagnostics (PRODI), Ruhr-University Bochum, Bochum, Germany; [5]Institut für Biologische Informationsprozesse, Molekular- und Zellphysiologie (IBI-1), Forschungszentrum Jülich, Jülich, Germany.

Supporting Information

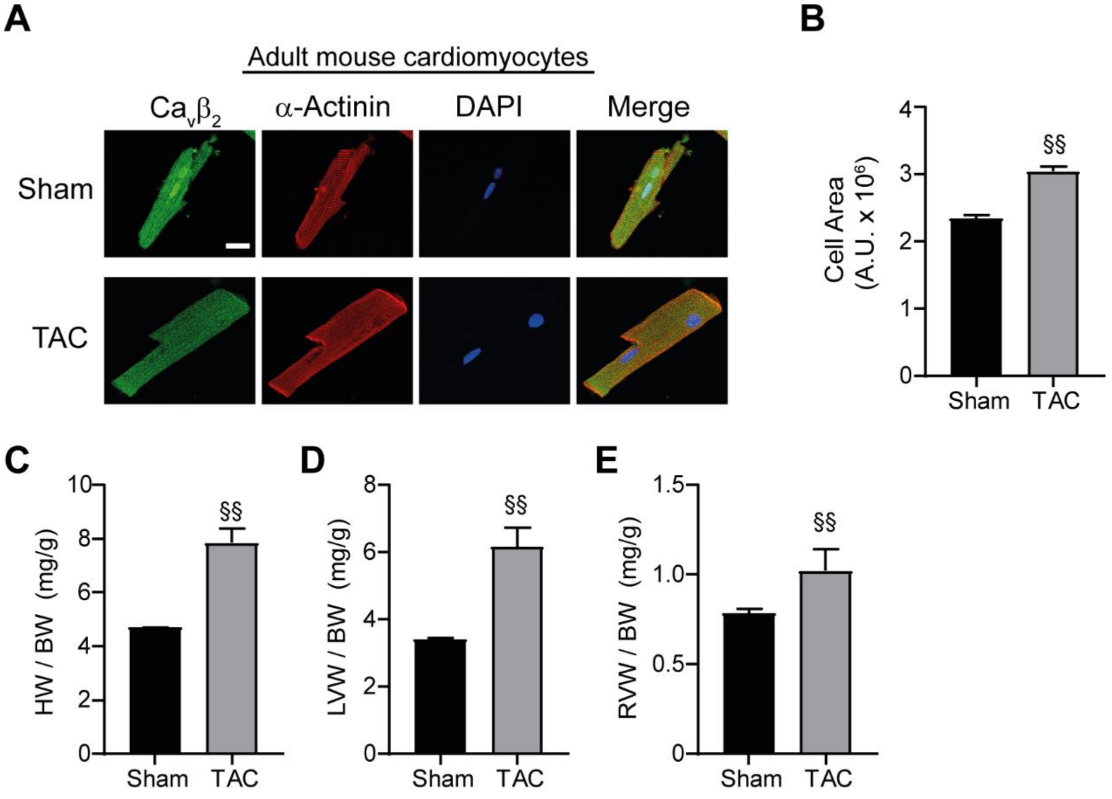

Figure S1. **Transverse aortic constriction induces left ventricle hypertrophy in adult mice.** (*A*) Confocal fluorescence images of representative adult mouse cardiomyocytes from sham- and TAC-operated mice stained for Ca$_v\beta_2$ (green), α-actinin (red) and nucleus (DAPI, blue). Scale bar represents 15 μm. (*B*) Bar plot of the mean values of cardiomyocyte cell area from sham-operated (n=280 cells) and TAC-operated mice (n=210 cells). Cells from 3 animals per group were measured. (*C-E*) B plots of the mean values of the the heart-to-body weight ratio (*C*), the left ventricle-to-body weight ratio (*D*) and the right ventricle-to-body weight ratio (*E*) from sham-operated (n=3) and TAC-operated mice (n=3). Mean ± SEM; §§ $p<0.01$ (two-tailed unpaired t-test).

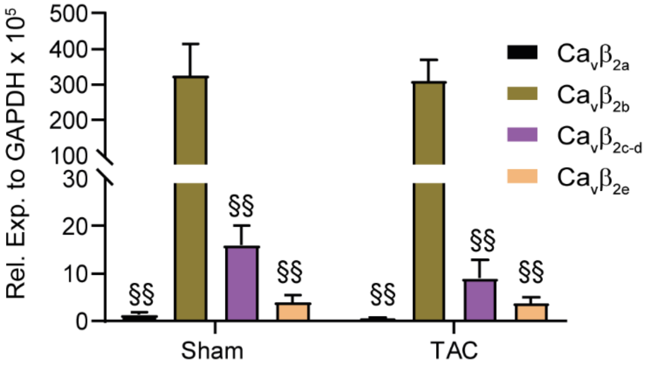

Figure S2. **$Ca_v\beta_2$ splice variants have similar expression in sham- and TAC-operated mice**. Bar plot of the qRT-PCR analyses of the expression of $Ca_v\beta_2$ splice variants in heart samples from sham- (n=3) and TAC-operated (n=3) mice. Mean ± SEM; §§ $p<0.01$ vs $Ca_v\beta_{2b}$ expression in the same group. (two-way ANOVA with Holm–Sidak´s method).
